# A 28 mK Resolution, −0.45 °C/+0.51 °C Inaccuracy Temperature Sensor Using Dual-Comparator Architecture and Logic-Controlled Counting Method

**DOI:** 10.3390/mi16080947

**Published:** 2025-08-18

**Authors:** Yubin Xu, Tongyu Luo, Lin Peng

**Affiliations:** 1 School of Electronics and Communication Engineering, Guangzhou University, Guangzhou 510006, China; 32207400088@e.gzhu.edu.cn (Y.X.); 32207400013@e.gzhu.edu.cn (T.L.); 2 Key Lab of Si-Based Information Materials & Devices and Integrated Circuits Design, Guangzhou University, Guangzhou 510006, China

**Keywords:** CMOS temperature sensor, wide temperature range, high resolution, high accuracy, bias circuit, dual-comparator structure, control logic

## Abstract

This paper presents an all-CMOS temperature sensor with low power consumption, wide temperature range, and high precision in a 180 nm CMOS process. Based on the I–V characteristics of MOSFETs in the subthreshold region and the negative exponential biasing current generated by the self-bootstrapped bias circuit, the proposed temperature-sensing front-end produces CTAT and PTAT voltages with high linearity and high sensitivity. The voltage-to-time converter (VTC) adopts a dual-comparator architecture to expand the time interval for improving resolution. The control logic unit is designed to count only within the time interval, eliminating interference during low-level periods and enhancing the accuracy of temperature measurement. The implemented sensor achieves an inaccuracy of −0.45 °C/+0.51 °C (3σ) from −40 °C to 130 °C after a two-point calibration with a resolution of 28 mK and consumes 503 nW at 27 °C when operating at 1 V, with an FoM of 7.9 pJ·K^2^.

## 1. Introduction

Temperature sensors play critical roles in on-chip thermal management [1,2,3], ambient temperature monitoring [4], biomedical devices [5,6,7], and other fields. In recent years, wireless sensor networks have been evolving toward lower power consumption, requiring sensors to perform precise temperature measurements under the constraints of small battery capacities. This trend has directly driven the development of temperature sensors toward lower power consumption, wider operating ranges, and higher measurement accuracy [8].

Up to now, many temperature sensors with different structures have been proposed. The base-emitter voltage of a bipolar junction transistor (BJT) exhibits excellent linearity with temperature, and this type of temperature sensor demonstrates outstanding performance in terms of operating range, resolution and accuracy. However, the inclusion of high-gain operational amplifiers, coupled with the requirement for substantial and stable bias currents, contributes to power consumption on the order of μW [9,10,11]. Resistance-based temperature sensors utilize the characteristic that the resistance of metal or semiconductor materials varies linearly with temperature to sense thermal changes, enabling excellent linearity and high precision [12,13,14]. However, challenges such as the high cost of metal materials, which limits large-scale deployment, and the potential introduction of self-heating effects due to external excitation, which may compromise measurement accuracy, remain significant issues.

Metal–Oxide–Semiconductor (MOS)-type temperature sensors exhibit significant advantages in terms of power consumption and footprint area. In [15], Wang et al. employed an ultra-low-power current reference generator to charge the MIM capacitor, and a least significant bit (LSB)-first algorithm-controlled capacitor charging time feedback loop was utilized to digitize the temperature. This approach achieve sub-nW power consumption but at the cost of reduced accuracy. In [16], using MOSFETs with different channel lengths, the subthreshold current ratio is converted to a frequency ratio, achieving low power and reduced corner dependency but with moderate accuracy. In [17], by leveraging the difference in the temperature-dependent threshold voltage slopes of two types of PMOS transistors for temperature sensing, this method eliminates the need for complex reference circuits, achieving moderate power consumption and accuracy. In summary, all-CMOS temperature sensors exhibit superior performance in terms of power consumption compared to BJT-based temperature sensors; however, achieving simultaneous optimization of both high resolution and high accuracy remains technically challenging.

In addition, there is a field of research on resonant temperature sensors based on the use of bulk acoustic waves and surface acoustic waves. These devices can operate at helium temperatures up to +900 °C. They do not require a power supply but rely on the piezoelectric effect and acoustic waves to function.

In this paper, we propose a fully CMOS biasing circuit. By generating a biasing current in the form of a negative exponential function, the circuit enables MOSFETs operating in the subthreshold region to produce proportional to absolute temperature (PTAT) and complementary to absolute temperature (CTAT) voltages with high linearity and sensitivity, thereby improving temperature sensing accuracy and resolution. In the voltage-to-time conversion circuit, a dual-comparator architecture is adopted to perform subtraction on PTAT and CTAT voltages, expanding the time interval to enhance resolution. In the time-to-digital conversion circuit, a control logic-based counting method is designed to eliminate interference during the counting process and improve temperature measurement accuracy.

The paper is organized as follows. The operation principle is discussed in Section 2. Then the circuit design is described in Section 3. In Section 4, the simulation results and the comparison with other published designs are presented. The conclusion is drawn in Section 5.

## 2. Proposed Temperature Sensor Circuit

### 2.1. Architecture

**Figure 1:**
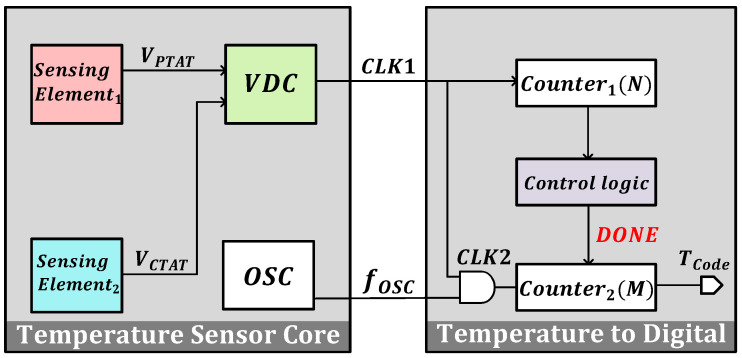
Block diagram of the proposed temperature-to-digital converter.

Figure 1 shows a block diagram of the proposed temperature-to-digital converter. The proposed temperature sensing element detects the temperature and outputs the PTAT voltage $V_{PTAT}$ and the CTAT voltage $V_{CTAT}$. The voltage-to-time converter (VTC) transforms the input voltages $V_{PTAT}$ and $V_{CTAT}$ into duty cycle signals while inheriting the property of temperature. ${Counter}_{1}$ is a programmable *N*-bit counter that performs continuous counting of the CLK1 signal to determine the number of time intervals (τ) and generate control signals. The oscillator generates a clock signal $f_{OSC}$ that undergoes a logical AND operation with the time interval (τ), thus converting the duty cycle information into an output clock signal (CLK2). ${Counter}_{2}$ is an *M*-bit counter. After ${Counter}_{1}$ completes the count of *N*-bits, the control logic unit outputs the stop signal DONE, and ${Counter}_{2}$ stops counting and outputs the digital code $T_{Code}$. The transitive relation can be expressed as

(1) $$\tau =\Delta t\cdot N=\frac{NC(V_{\mathrm{PTAT}}-V_{C\mathrm{TAT}})}{I}\propto T,$$

where *N* is the number of digits of ${Counter}_{1}$ and *C* is the capacitance value. The final output digital code of the temperature sensor can be expressed as

(2) $$T_{Code}=f_{OSC}\cdot \tau =f_{OSC}\cdot \Delta t\cdot N.$$

In Equation (2), it can be observed that within the target temperature range, the linearity of the temperature-to-digital converter primarily depends on the temperature-sensing front-end and the oscillator frequency.

### 2.2. Operating Principle

**Figure 2:**
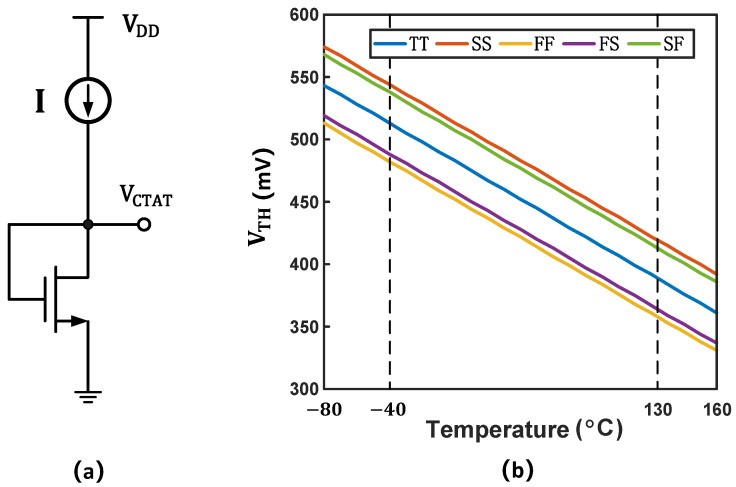
(**a**) Scheme of principle of a simple $V_{CTAT}$ reference. (**b**) Threshold voltage of NMOS transistor.

In temperature sensing elements, BJT-type temperature sensing elements exhibit excellent accuracy and a wide temperature range, while MOS-type temperature sensing elements offer superior low-voltage and low-power-consumption characteristics. As shown in Figure 2a, a voltage inversely proportional to the absolute temperature can be represented by a current source and an NMOS operating in the subthreshold region.

The drain current of a subthreshold-operated MOSFET is characterized by

(3) $$I_{D}=\left(\frac{W}{L}\right)I_{S}e^{\frac{\left(V_{GS}-V_{TH}\right)}{nV_{T}}}\left(1-e^{\frac{-V_{DS}}{V_{T}}}\right),$$

(4) $$I_{S}=\mu \left(n-1\right)C_{ox}{V_{T}}^{2},$$

where μ represents carrier mobility in the channel, $C_{ox}$ denotes the oxide capacitance per unit area, *W* and *L* correspond to the transistor’s width and length respectively, while *n* signifies the subthreshold swing coefficient. The terms $V_{TH}$ and $V_{GS}$ indicate threshold voltage and gate-to-source voltage, respectively. $V_{T}=\frac{KT}{q}$ is the thermal voltage, where *K* is the Boltzmann constant, *q* represents the elementary charge, and *T* stands for absolute temperature.

When $V_{GS}\geq 4V_{T}$, $I_{D}$ becomes virtually independent of $V_{DS}$, and thus $I_{D}$ can be modified as

(5) $$I_{D}=\left(\frac{W}{L}\right)I_{S}e^{\frac{\left(V_{GS}-V_{TH}\right)}{nV_{T}}}.$$

Therefore, $V_{GS}$ can be expressed as

(6) $$V_{GS}=nV_{T}\ln M+V_{TH},$$

(7) $$M=\frac{I_{D}}{I_{S}\left(\frac{W}{L}\right)}.$$

We note that $V_{T}\left(T\right)$, $V_{TH}\left(T\right)$ and μ(T) all exhibit temperature dependence. It is commonly assumed that the expression for $V_{TH}$ is [18]

(8) $$V_{TH}=V_{TH}\left(T_{0}\right)+\left(k_{t1}+k_{t2}V_{BS}\right)\left(\frac{T}{T_{0}}-1\right),$$

where $V_{TH}\left(T_{0}\right)$ is the threshold voltage at the reference temperature ($T_{0}≅300$ K), and $V_{BS}$ is the body voltage to the source of the transistor. The temperature coefficients $k_{t1}$ and $k_{t2}$ are negative.

As shown in Figure 2b, the threshold voltage of the NMOS transistor exhibits excellent CTAT characteristics over a wide temperature range. Therefore, to ensure that $V_{GS}$ also demonstrates good CTAT characteristics, the $nV_{T}\ln M$ term in Equation (6) must exhibit strong CTAT behavior.

We assume the bias current has the general form [19]

(9) $$I\left(T\right)=\alpha \mu T^{2}f\left(T\right),$$

where α is a temperature-invariant constant. By substituting Equations (8) and (9) into $V_{GS}=nV_{T}\ln M+V_{TH}$ and taking the first-order derivative of $V_{GS}$ with respect to temperature, we obtain

(10) $$\begin{array}{c} \frac{\partial V_{GS}}{\partial T}=\frac{k_{t1}}{T_{0}}+n\frac{K}{q}\ln\left(\frac{\alpha }{S{\left(\frac{k}{q}\right)}^{2}}\right)+n\frac{K}{q}\left[\ln\left(f\left(T\right)\right)+\frac{T}{f\left(T\right)}\frac{\partial f\left(T\right)}{\partial T}\right], \end{array}$$

where $S=\left(n-1\right)C_{ox}\left(\frac{W}{L}\right)$. To achieve a highly linear $V_{GS}$, the term inside the brackets must remain invariant with temperature. Therefore, the expression form of the current can be further assumed as [19]

(11) $$I_{D}\left(T\right)=\alpha \mu T^{2}e^{\left(\frac{AT+B}{CT}\right)},$$

where *A*, *B*, *C* are temperature-invariant constants. The current is assumed to follow an exponential form because this type of bias current can be derived from the subthreshold current of a MOSFET. Substituting Equation (11) into Equation (10), we obtain

(12) $$\frac{\partial V_{GS}}{\partial T}=\frac{k_{t1}}{T_{0}}+n\frac{K}{q}\ln\left(\frac{\alpha }{S{\left(\frac{k}{q}\right)}^{2}}\right)+n\frac{KA}{qC}.$$

As can be seen from Equation (12), $\frac{\partial V_{GS}}{\partial T}$ does not contain a temperature-dependent term. Therefore, when the bias current follows the form given in Equation (11), $V_{GS}$ exhibits excellent linearity.

To achieve the CTAT characteristic for $V_{GS}$, it is necessary to ensure $\frac{\partial V_{GS}}{\partial T}<0$. From the above derivation, it can be concluded that $k_{t1}$ is negative. For the term $n\frac{K}{q}\ln\left(\frac{\alpha }{S{\left(\frac{k}{q}\right)}^{2}}\right)$, it can be simplified through straightforward calculation to $Dn+n\frac{K}{q}\ln\left(\frac{\alpha }{S}\right)$, where *D* has a magnitude of approximately 0.002. Thus, the Dn term can be neglected. Consequently, the CTAT behavior of $V_{GS}$ is primarily determined by $\frac{\alpha }{S}$ and $\frac{A}{C}$. Based on this analysis, a more precise expression for the bias current should take the form of a negative exponential current.

In this section, we demonstrate how the assumed form of bias current enables an NMOS transistor operating in the subthreshold region to generate a CTAT voltage, while simultaneously analyzing how the bias current formulation affects voltage linearity. The subsequent section will detail the implementation of the bias circuit and present error analysis for both CTAT and PTAT reference voltages.

## 3. Circuit Implementation

### 3.1. Overall Circuit Schematic

**Figure 3:**
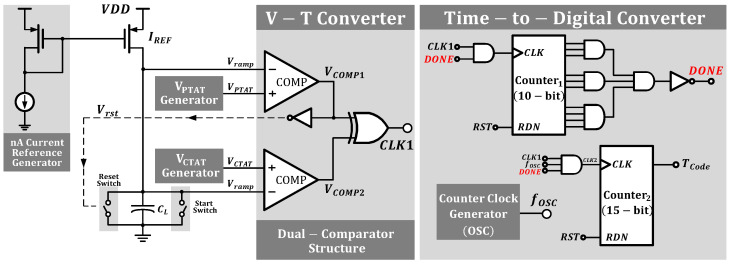
Overall circuit schematic of the proposed temperature sensor.

Figure 3 shows the general circuit schematic of the proposed temperature sensor. The design consists of three parts: a temperature sensing element, a voltage-to-time conversion circuit, and a time-to-digital conversion circuit. Each part will be described in detail in the following sections.

### 3.2. Temperature Sensing Element

**Figure 4:**
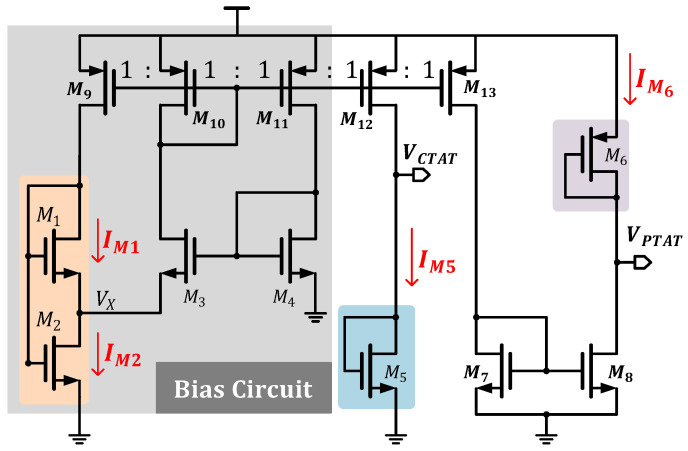
Schematic of the $V_{CTAT}$ and $V_{PTAT}$ voltage generator.

Figure 4 illustrates the proposed $V_{CTAT}$ and $V_{PTAT}$ voltage generation circuits, both utilizing the same biasing circuit. Similar to [20], this biasing circuit adopts an all-CMOS structure. The entire circuit operates at a low supply voltage of 1 V, where $M_{1}$ and $M_{2}$ are implemented as self-cascoded MOSFETs (SCMs). This structure replaces traditional resistors to meet the requirements for low-voltage and low-current operation [21].

In the bias circuit, $M_{9}$-$M_{10}$ form a current mirror with ${\left(W/L\right)}_{9}/{\left(W/L\right)}_{10}=1$, resulting in $I_{M1}=\frac{1}{2}I_{M2}$. Meanwhile, $M_{1}$ and $M_{2}$ operate in the subthreshold region. Considering the impact of $V_{BS}$ on the threshold voltage, the threshold voltage of $M_{1}$ is corrected as [20]

(13) $$V_{TH1}^{-}=V_{TH1}+\left(n-1\right)V_{X}.$$

Combining $I_{M1}=\frac{1}{2}I_{M2}$ and Equation (13), we obtain

(14) $$\begin{array}{c} {\left(\frac{W}{L}\right)}_{1}I_{S}e^{\frac{\left(V_{G1}-V_{X}-V_{TH1}^{-}\right)}{nV_{T}}}\;=\frac{1}{2}{\left(\frac{W}{L}\right)}_{2}I_{S}e^{\frac{\left(V_{G2}-V_{TH2}\right)}{nV_{T}}}\left(1-e^{\frac{-V_{X}}{V_{T}}}\right). \end{array}$$

Since $V_{G1}=V_{G2}$ and $V_{TH1}\approx V_{TH2}$, Equation (14) can be simplified to

(15) $$V_{X}=V_{T}\ln\left(\frac{{2\left(\frac{W}{L}\right)}_{1}}{{\left(\frac{W}{L}\right)}_{2}}+1\right).$$

Additionally, the current flowing through $M_{1}$ can be expressed as

(16) $$I_{M1}=\frac{1}{2}I_{S}{\left(\frac{W}{L}\right)}_{2}e^{\frac{\left(V_{GS2}-V_{TH2}\right)}{nV_{T}}}\left(1-e^{\frac{-V_{X}}{V_{T}}}\right).$$

**Figure 5:**
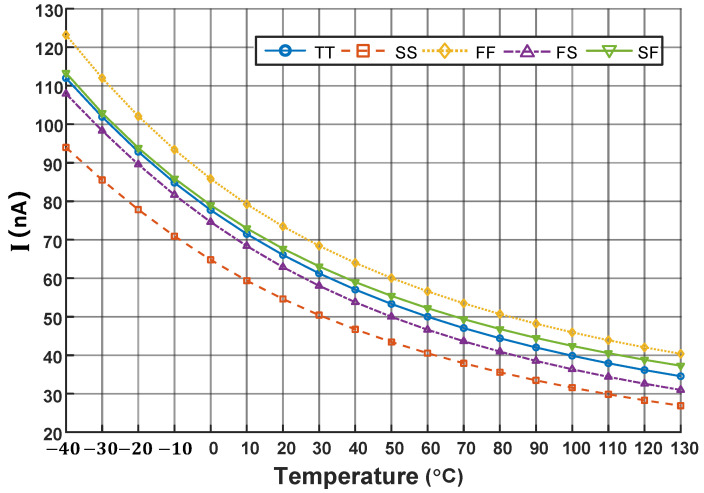
Current generated by bias circuit across process corners.

Substituting Equation (15) into Equation (16), we obtain

(17) $$I_{M1}=I_{S}\frac{K_{1}K_{2}}{{2K}_{1}+K_{2}}e^{\frac{\left(V_{GS2}-V_{TH2}\right)}{nV_{T}}},$$

where $K_{1}={\left(\frac{W}{L}\right)}_{1}$ and $K_{2}={\left(\frac{W}{L}\right)}_{2}$. Based on Equation (8), the relationship between the threshold voltage and temperature can be determined. From Figure 5, it can be observed that the bias current exhibits a negative exponential function form across all process corners. Therefore, we conclude that the bias circuits in Figure 4 generate a current of the form given by Equation (11).

**Figure 6:**
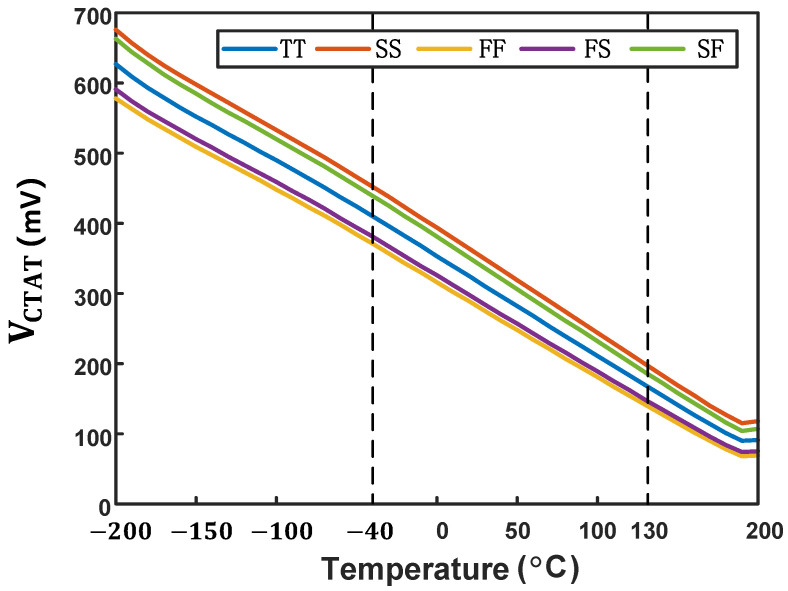
Simulation results of $V_{CTAT}$ across process corners.

**Figure 7:**
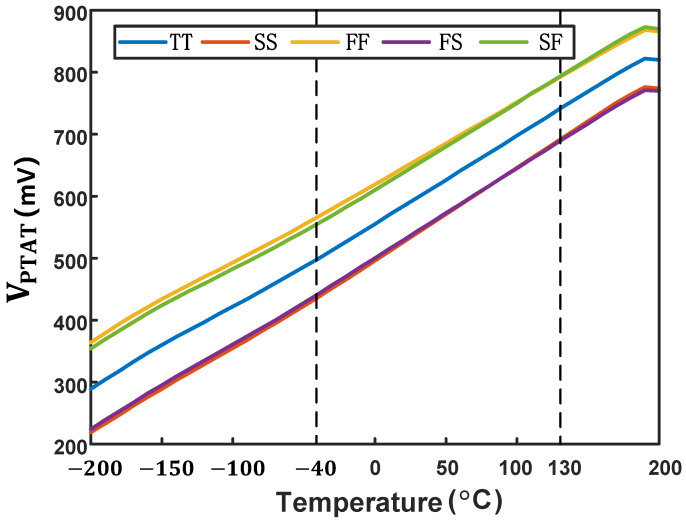
Simulation results of $V_{PTAT}$ across process corners.

As shown in Figure 6 and Figure 7, we simulated the $V_{CTAT}$ and $V_{PTAT}$ characteristics across a wide temperature range. The simulation results indicate that both $V_{CTAT}$ and $V_{PTAT}$ maintain excellent linearity from −40 °C to 130 °C, fully satisfying the temperature-sensing requirements for on-chip thermal management applications [9].

Since $I_{M5}/I_{M1}={\left(W/L\right)}_{12}/{\left(W/L\right)}_{9}=1$, by combining Equations (8) and (17), the first-order derivative of $V_{CTAT}$ with respect to temperature can be derived as

(18) $$\frac{\partial V_{CTAT}}{\partial T}=\frac{k_{t1}}{T_{0}}+n\frac{K}{q}\ln\left[\frac{K_{1}K_{2}M}{\left({2K}_{1}+K_{2}\right)K_{5}}\right].$$

Since $k_{t1}$ is negative, by carefully designing the width-to-length ratios of $M_{1}$ and $M_{2}$, the first-order partial derivative of $V_{CTAT}$ with respect to temperature is engineered to be negative, ensuring $V_{CTAT}$ exhibits a negative temperature coefficient (NTC).

In the $V_{PTAT}$ voltage generation circuit shown in Figure 4, the bias circuit is identical to that of the $V_{CTAT}$ generation circuit. The PTAT voltage can be expressed as

(19) $$V_{PTAT}=V_{DD}-V_{SG6}.$$

From the preceding analysis, it is demonstrated that the source-gate voltage of $M_{6}$ also exhibits a negative temperature coefficient (NTC). By performing the $V_{DD}-V_{SG6}$ operation, the CTAT characteristic can be converted to a PTAT characteristic. However, this introduces a power supply voltage term in Equation (19), causing $V_{PTAT}$ to vary with supply voltage fluctuations, thereby compromising the power supply rejection (PSR) of the temperature-sensing front-end.

To achieve better resolution, the temperature-sensing front-end needs to provide as wide a voltage variation range as possible. By examining Equation (18), we observe that the width-to-length ratio of $M_{1}$ and $M_{2}$ serves as the influencing factor *m*. Adjusting *m* can effectively expand the voltage range.

**Figure 8:**
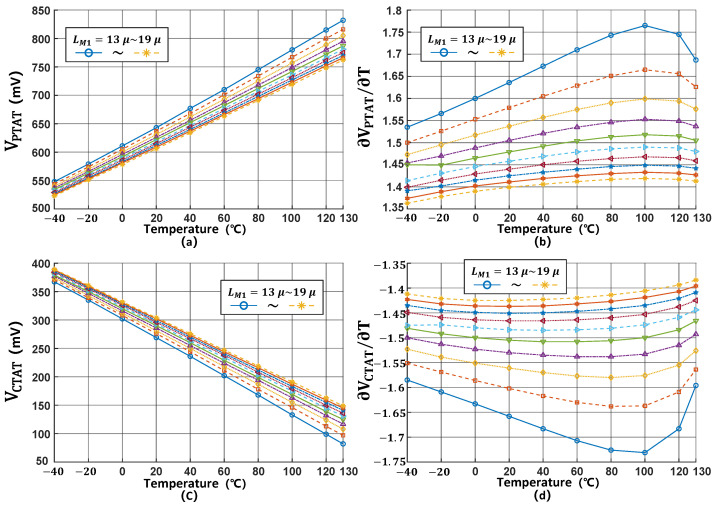
(**a**–**d**) $K_{1}$ variation effects on $V_{PTAT}$ and $V_{CTAT}$.

Figure 8 demonstrates the variations in $V_{PTAT}$ and $V_{CTAT}$ voltages and their corresponding slopes as $L_{M1}$ is adjusted from 13 μm to 19 μm. The simulation results demonstrate that increasing $L_{M1}$ expands the voltage range of both $V_{PTAT}$ and $V_{CTAT}$, but at the cost of degrading their linearity performance.

**Figure 9:**
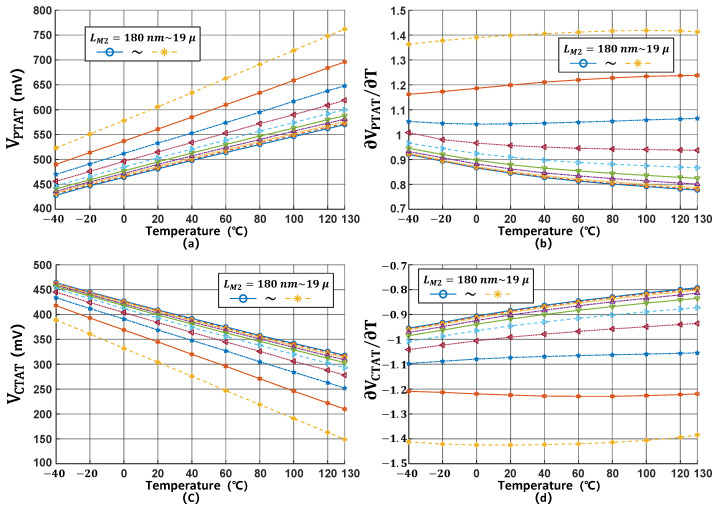
(**a**–**d**) $K_{2}$ variation effects on $V_{PTAT}$ and $V_{CTAT}$.

Similarly, Figure 9 demonstrates the impact of $L_{M2}$ variations ranging from 180 nm to 19 μm on both $V_{PTAT}$ and $V_{CTAT}$. In this case, increasing $L_{M2}$ simultaneously expands the voltage range and improves voltage linearity.

**Figure 10:**
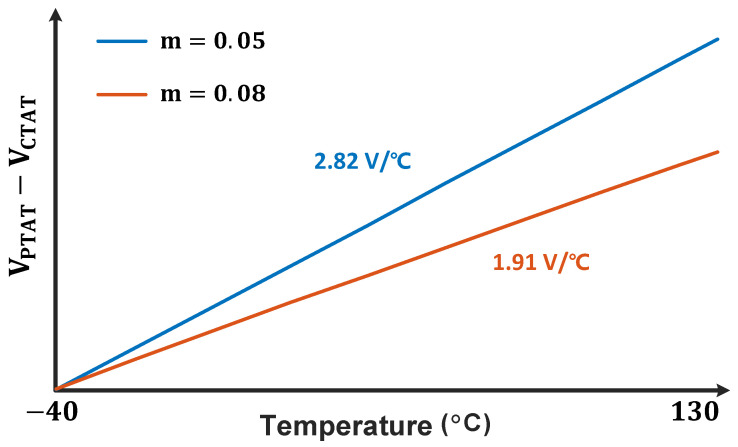
Slope variation of $V_{PTAT}-V_{CTAT}$.

According to Equation (1), the performance of the proposed temperature sensor is closely related to $V_{PTAT}-V_{CTAT}$. While meeting the requirements of the voltage-to-time converter, the sensitivity of $V_{PTAT}-V_{CTAT}$ can be enhanced by adjusting the scaling factor *m*, as demonstrated in Figure 10.

**Figure 11:**
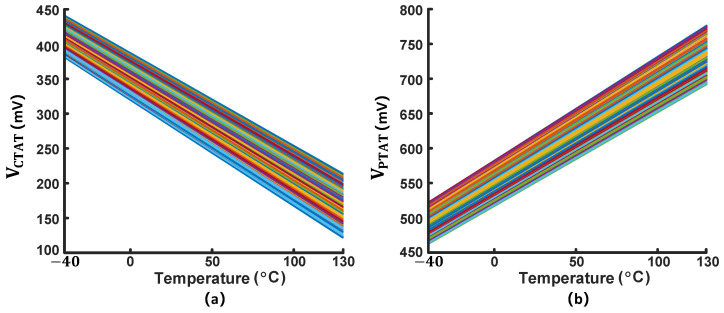
(**a**) $V_{CTAT}$ and (**b**) $V_{PTAT}$ in 200-run Monte Carlo simulation, including both process variation and mismatch.

At the selected Taylor temperature, to verify the robustness of the process, Figure 11 demonstrates the data from 200-run Monte Carlo simulation of $V_{CTAT}$ and $V_{PTAT}$.

**Figure 12:**
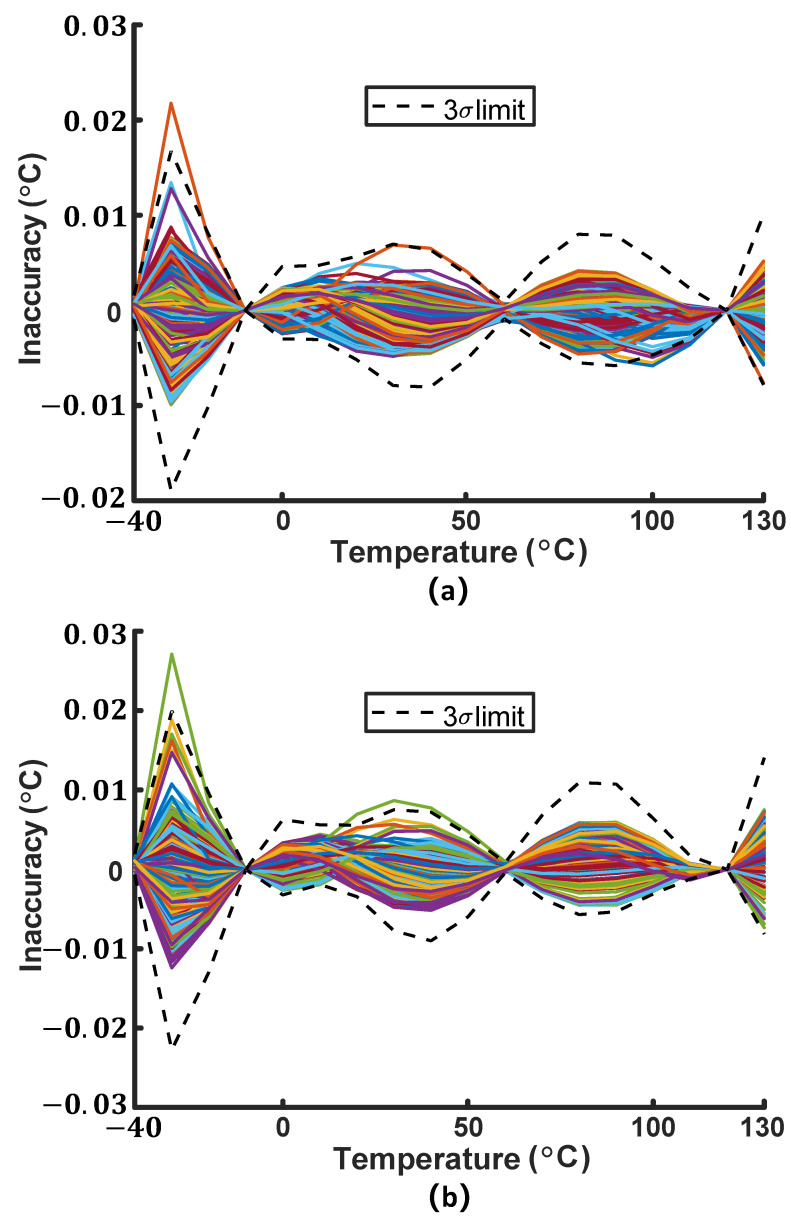
(**a**) Inaccuracy of $V_{CTAT}$ and (**b**) inaccuracy of $V_{PTAT}$ in 200-run Monte Carlo simulation, including both process variation and mismatch.

As shown in Figure 12, simulation results shows that after calibrating $V_{CTAT}$ and $V_{PTAT}$ at −10 °C and 120 °C, the error of both $V_{CTAT}$ and $V_{PTAT}$ is less than ±0.03 °C.

**Figure 13:**
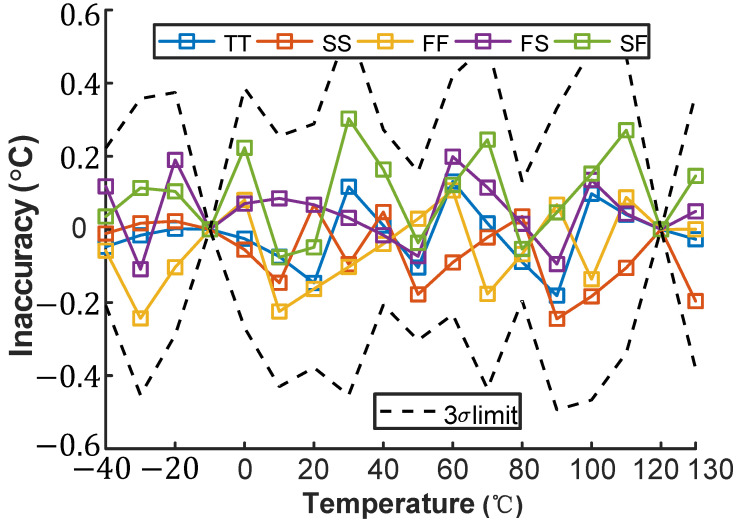
Temperature dependence of SE inaccuracy across process corners.

**Figure 14:**
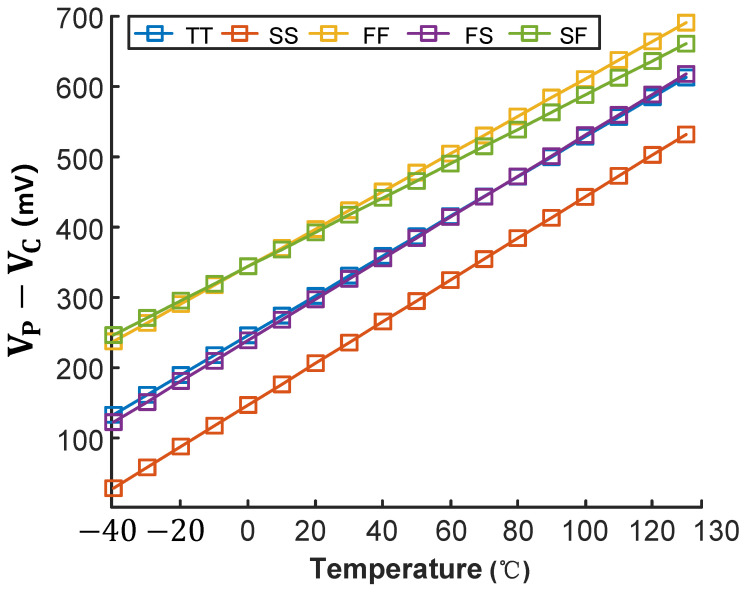
Temperature dependence of SE output across process corners.

Figure 13 and Figure 14 simulate the inaccuracy of the $V_{PTAT}-V_{CTAT}$ voltage across process corners in the temperature-sensing front-end under maximum voltage sensitivity conditions, operating from −40 °C to 130 °C. After two-point calibration and nonlinearity correction, the $V_{PTAT}-V_{CTAT}$ voltage exhibits errors below ±0.5 °C across all process corners.

### 3.3. Voltage-to-Time Converter

**Figure 15:**
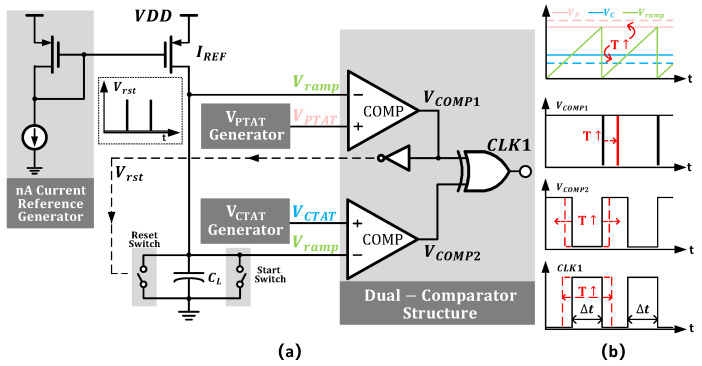
(**a**) Detailed of the voltage-to-time converter. (**b**) Output waveform of the key node.

Figure 15a illustrates the details of the voltage-to-time converter (VTC), the circuit designed to transform voltage into time intervals. According to Equation (1), the VTC should have excellent linearity to maintain the high linearity of the SE. The circuit operates as follows: Initially, the start switch resets $V_{ramp}$ to zero. $V_{PTAT}$ and $V_{CTAT}$ are fed into two comparators. When $V_{ramp}$ exceeds $V_{CTAT}$, comp 2’s output ($V_{COMP2}$) toggles; when $V_{ramp}$ surpasses $V_{PTAT}$, comp 1’s output ($V_{COMP1}$) toggles, activating the reset switch via an inverter to discharge the capacitor and begin a new cycle. However, at the end of a cycle, the capacitor discharges to zero, and the comparator requires the next rising edge to trigger a comparison, causing $V_{COMP1}$ to return to a high level. Therefore, the corrected time interval should be

(20) $$\Delta t^{*}=\Delta t+t_{CLK}.$$

where $t_{CLK}$ represents the single clock cycle of the comparator. This issue is mitigated by incorporating control logic.

The output waveforms of key nodes in the VTC are shown in Figure 15b. As the temperature rises, the dual-comparator architecture produces a highly sensitive $V_{PTAT}-V_{CTAT}$ term that expands the voltage range, resulting in wider time intervals for higher resolution.

### 3.4. Time-to-Digital Converter

**Figure 16:**
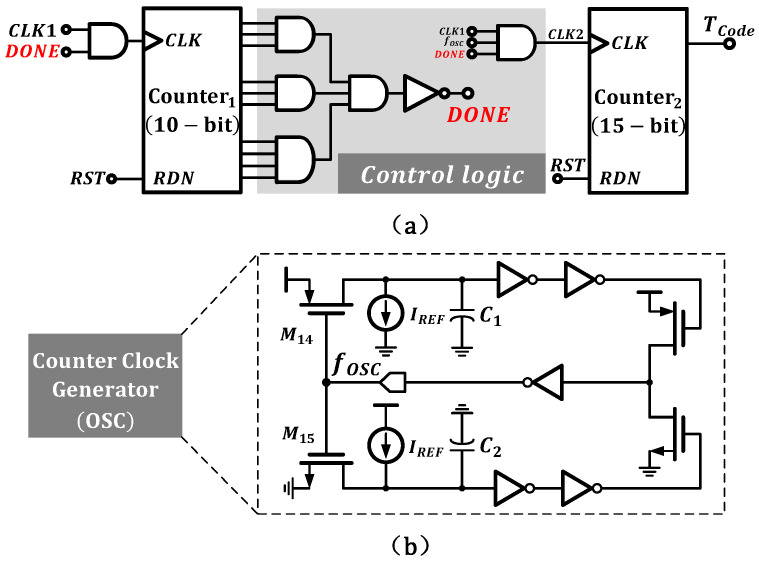
(**a**) Control logic unit. (**b**) Schematic of relaxation oscillator.

Figure 16 details the time-to-digital converter (TDC), where Figure 16a shows the control logic unit. Composed of AND and NOT gates, the control logic connects to the output of Counter 1. When Counter 1 reaches its maximum count, the DONE signal transitions from high to low, halting Counter 2. The count value N of Counter 1 represents the number of time intervals recorded from the VTC output. According to Equation (1), Δt inherits temperature-dependent characteristics, while the low-level periods between adjacent Δt intervals exhibit nonlinearity. The proposed control logic extracts the linear Δt intervals and counts only within them, ignoring the nonlinear interference from the low-level periods, thereby improving precision.

**Figure 17:**
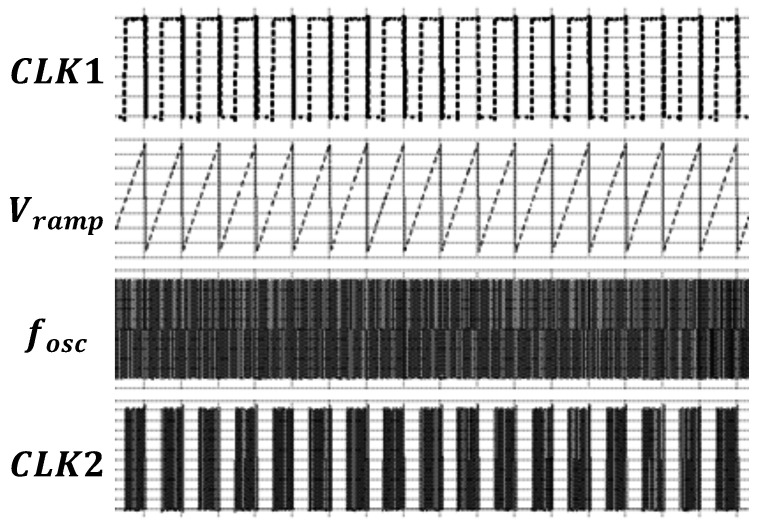
Output waveform of the frequency-to-digital converter.

As shown in Figure 17, CLK1 is the clock signal generated by the VTC, and $V_{ramp}$ reflects the voltage variation across the capacitor $C_{L}$. $f_{OSC}$ is the output clock signal of the relaxation oscillator. Under the control of the logic unit, CLK2 is generated, enabling counting only within Δt. Regarding the previously mentioned $t_{CLK}$ issue, since counting only occurs within Δt and the time interference remains consistent at each temperature, its impact on resolution and accuracy can be ignored.

In the time-to-digital conversion circuit, a temperature-insensitive ideal reference frequency is required to achieve digital conversion of temperature characteristics. As shown in Figure 16b, the oscillator we employed is similar to [22] and was specifically designed for low-power operation.

The oscillator consists of two branches, with each branch controlling half of the oscillation period through charging and discharging a capacitor using current $I_{REF}$. Compared with conventional single-branch oscillators [23], this architecture eliminates capacitor reset nonlinear interference. The oscillator frequency $f_{OSC}$ can be expressed by the following equation:

(21) $$f_{OSC}=\frac{I_{REF}}{C_{cap}\cdot V_{DD}}.$$

**Figure 18:**
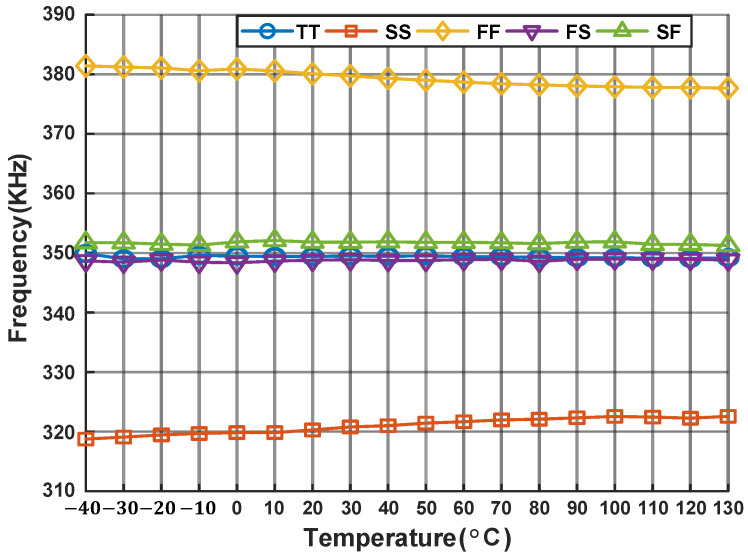
Output frequencies of relaxation oscillator.

**Figure 19:**
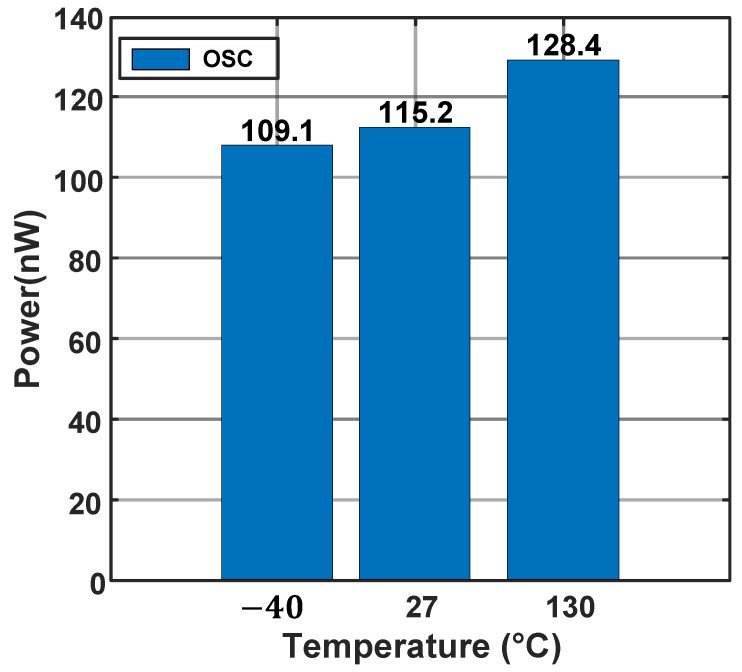
Power consumption of the oscillator at −40 °C, 27 °C and 130 °C.

Figure 18 illustrates the variation in the relaxation oscillator’s frequency across different process corners. The maximum frequency deviation is approximately 3 kHz under the SS process corner and around 4 kHz under the FF process corner. In all other process corners, the oscillator maintains excellent temperature stability. Figure 19 demonstrates the power consumption of the proposed oscillator at −40 °C, 27 °C and 130 °C.

## 4. Simulation Results

**Figure 20:**
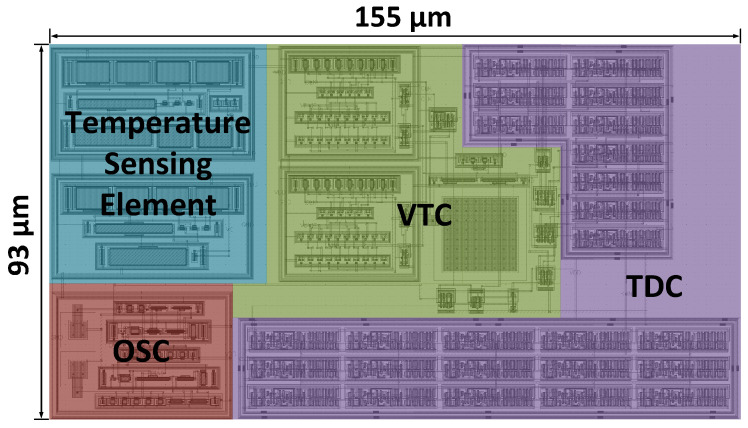
Layout of the proposed temperature sensor.

The proposed temperature sensor circuit has been successfully impS202411078034imulated results are plotted in Figure 20 with a total area of 0.015 mm^2^. In this work, temperature range, resolution, and accuracy are key performance indicators.

**Figure 21:**
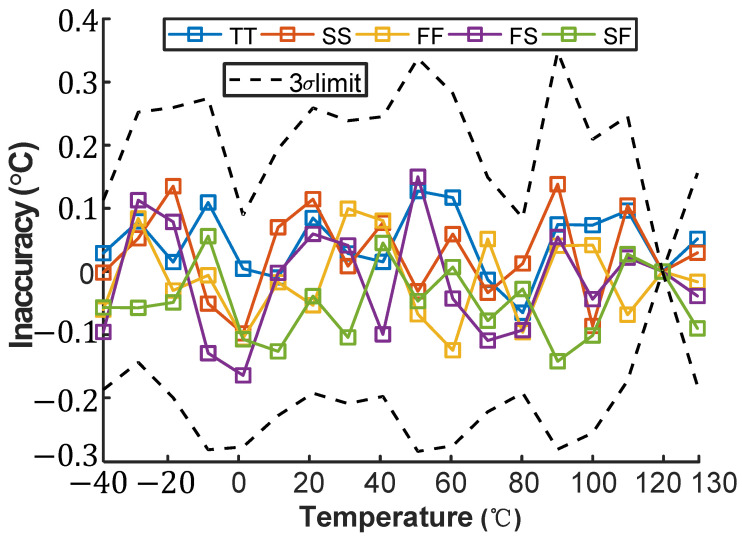
Temperature error of the proposed temperature sensor with one-point calibration and systematic nonlinearity removal.

**Figure 22:**
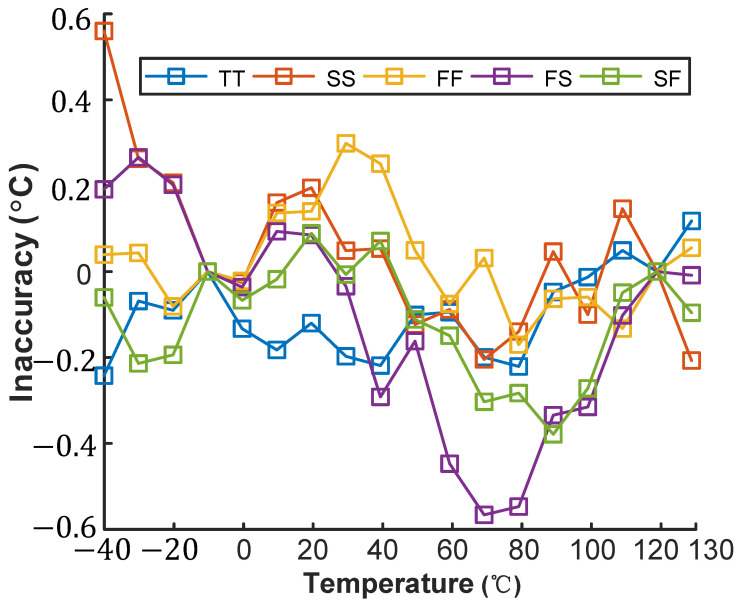
Temperature error of the proposed temperature sensor with two-point calibration.

The temperature-sensing accuracy of the designed temperature sensor is influenced by the layout parasitic components. This is because the voltage-to-time conversion circuit employs a method of charging and discharging the capacitor to transform the voltage’s temperature characteristics into the temperature characteristics based on time. The presence of parasitic capacitance affects the value of the designed capacitance, which in turn alters the temperature characteristics inherited by the time interval, ultimately leading to a decrease in sensing accuracy. Figure 21 shows the error of the proposed temperature sensor after calibration at one point and removal of nonlinearity. As shown in Figure 22, the maximum error of the proposed temperature sensor after calibration at two points is 1.2 °C.

**Figure 23:**
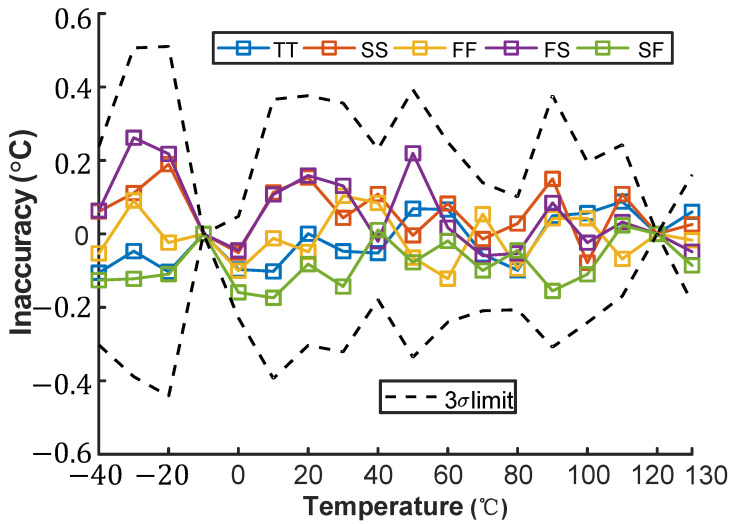
Temperature error of the proposed temperature sensor with two-point calibration and systematic nonlinearity removal.

This paper uses third-order polynomial fitting to remove systematic errors through a two-point calibration method. As shown in Figure 23, using −10 °C and 120 °C as reference points and after removing nonlinearity, the maximum measurement error was reduced to −0.45 °C/+0.51 °C.

**Figure 24:**
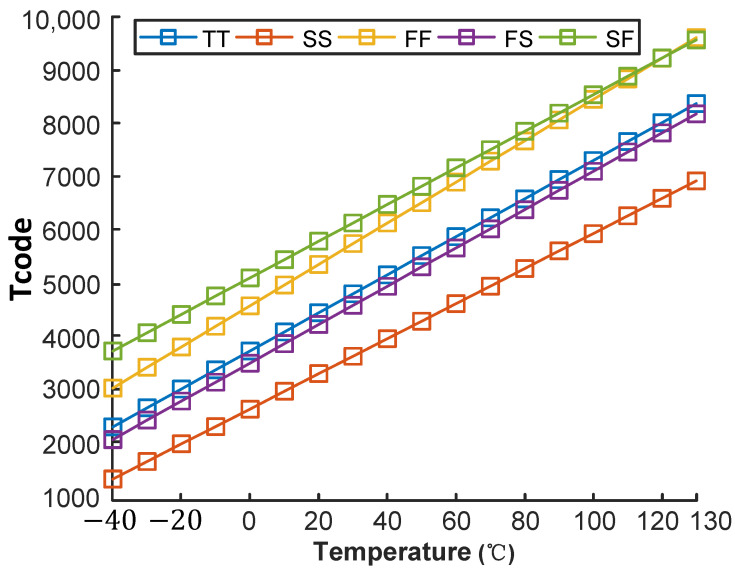
Output temperature code.

The resolution of a temperature sensor refers to the smallest temperature change that the sensor can detect, which is the minimum temperature fluctuation that can cause a change in the least significant bit (LSB) of the digital output code. Figure 24 presents the output temperature codes (Tcode) of the proposed temperature sensor, and the Tcode range is 2274–8386 at a typical corner. The code range is approximately 6112, corresponding to a resolution of 28 mK.

**Figure 25:**
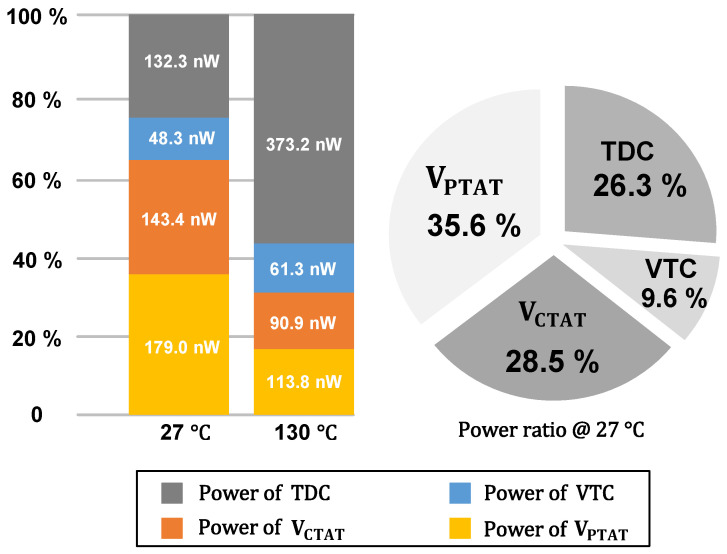
Power consumption of the oscillator at 27 °C and 130 °C.

Figure 25 illustrates the overall power consumption distribution of the proposed temperature sensor at 27 °C and 130 °C. The bar chart displays the power consumption of various components of the temperature sensor, with the power consumed by the oscillator included within the TDC, as detailed in Figure 19. The pie chart represents the percentage of power consumption attributed to each component of the temperature sensor at 27 °C, with the temperature sensing circuit accounting for 64.1% of the total power consumption.

**Figure 26:**
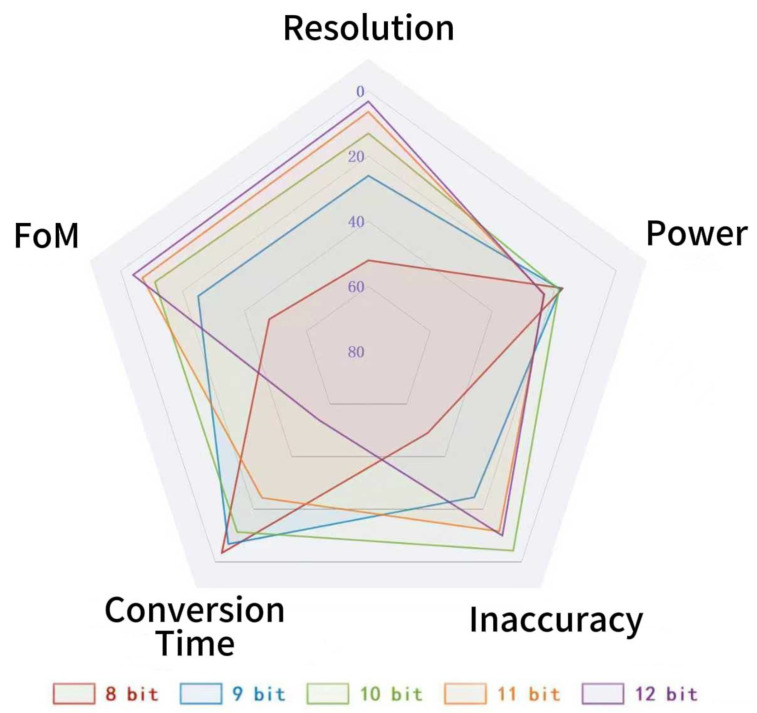
Performance comparison diagram under different counter bit widths.

As observed in Figure 26 and consistent with the previous discussion, the bit-width of Counter 1 significantly impacts the performance of the proposed temperature sensor. Increasing Counter 1’s bit width enhances resolution, but this improvement is achieved at the expense of degraded accuracy, elevated power consumption, and prolonged conversion time.

Table 1 shows the comparison of this design with other temperature sensors. This design achieves outstanding resolution performance through the proposed dual-comparator structure, which expands the voltage range by constructing a $V_{PTAT}-V_{CTAT}$ term, thereby enhancing resolution. Furthermore, the design demonstrates competitive accuracy through two key innovations: (1) the temperature-sensing front-end employs negative-exponential current to improve the linearity of both PTAT and CTAT voltages, and (2) a control-logic-based counting method eliminates nonlinear interference during low-voltage periods. These techniques collectively enhance temperature measurement precision. The design achieves a wide temperature sensing range of −40 °C to 130 °C while maintaining an excellent resolution FoM.

## 5. Conclusions

In this paper, we presents a low-power, wide-temperature-range, and high-precision temperature sensor. The biasing circuit generates a biasing current in the form of a negative exponential function, enabling MOSFETs operating in the subthreshold region to produce CTAT and PTAT voltages with high linearity and high sensitivity. The readout circuit employs a dual-comparator structure and control logic-based counting method to enhance resolution and improve accuracy. Implemented in a 180 nm CMOS process, the sensor consumes 503 nW at a 1 V supply voltage. After two-point calibration and nonlinearity correction over the −40 °C to 130 °C range, it achieves a peak-to-peak error of −0.45 °C/+0.51 °C. Simulation results demonstrate that the proposed temperature sensor attains a resolution of 28 mK with a resolution FoM of 7.9 pJ·K^2^.

## Figures and Tables

**Table 1:** Performance summary and comparison with other works.

	This Work	JSSC [24]	TCAS-I [17]	TCAS-I [16]	MDPI [25]	APCCAS [26]	TCAS-II [27]
Year	2025	2019	2021	2023	2024	2022	2023
Technology (nm)	180	180	130	180	180	180	28
Transducer type	MOS	MOS	MOS	MOS	MOS	BJT	RES
Result	Post-Sim	Mea	Mea	Mea	Sim	Sim	Mea
Area (mm^2^)	0.015	0.074	0.07	0.055	N/A	N/A	0.0092
Temperature Range (°C)	−40–130	−20–80	0–80	0–100	0–120	−40–125	−40–100
Calibration	2-point	2-point	2-point	2-point	2-point	1-point	2-point
Inaccuracy (°C)	−0.45/+0.51	−0.9/+1.2	−0.4/+0.44	−0.5/+0.4	−0.38/+0.43	−0.3/+0.3	−0.62/+0.81
Relative Inaccuracy ^a^ (%)	0.57	2.1	1.05	0.9	0.675	0.36	1.02
Supply Voltage (V)	1	0.8	0.95	1	1.2	1	0.9
Power (nW)	503	11	196	20	1480	14500	123500
Conversion Time (ms)	20	839	59	50	26	0.204	0.404
Energy/Conversion (nJ)	10.06	8.9	11.56	1	38.39	2.96	49.9
Resolution (mK)	28	145	100	120	7.1	70	56.5
Resolution FoM ^b^ $\left(\mathrm{pJ}\cdot K^{2}\right)$	7.9	190	120	14.4	1.9	14.5	159

^a^ (Max Inaccuracy – Min Inaccuracy)/Temperature Range × 100. ^b^ Energy/Conversion × Resolution^2^.

## Data Availability

Data will be made available on request.
